# The Effect of Novel Synthetic Methods and Parameters Control on Morphology of Nano-alumina Particles

**DOI:** 10.1186/s11671-016-1472-z

**Published:** 2016-05-21

**Authors:** Yadian Xie, Duygu Kocaefe, Yasar Kocaefe, Johnathan Cheng, Wei Liu

**Affiliations:** Department of Applied Science, University of Quebec at Chicoutimi (UQAC), Chicoutimi, Quebec G7H2B1 Canada; Civil, Architectural & Environmental Engineering (CAEE), Drexel University, Philadelphia, PA 19104 USA; School of Architectural & Material Engineering, Guizhou Normal University, Guiyang, Guizhou 550001 China

**Keywords:** Alumina, Particle morphology, Reaction mechanism, Calcination method

## Abstract

Alumina is an inorganic material, which is widely used in ceramics, catalysts, catalyst supports, ion exchange and other fields. The micromorphology of alumina determines its application in high tech and value-added industry and its development prospects. This paper gives an overview of the liquid phase synthetic method of alumina preparation, combined with the mechanism of its action. The present work focuses on the effects of various factors such as concentration, temperature, pH, additives, reaction system and methods of calcination on the morphology of alumina during its preparation.

## Review

### Introduction

**Fig. 1:**
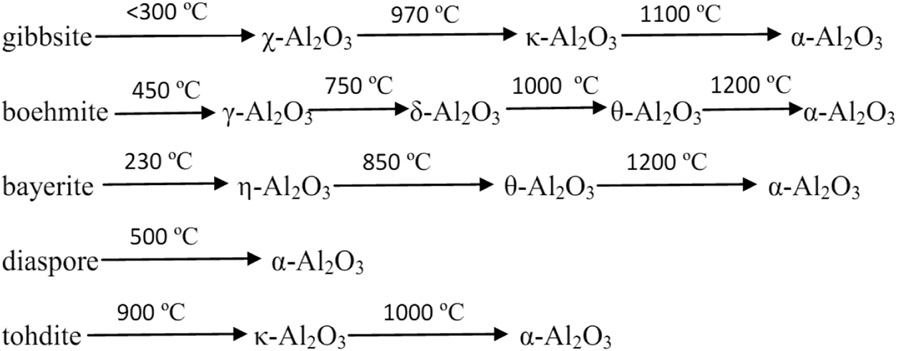
Phase transformation of alumina

Different materials are essential for the social development. Generally, certain material structure and morphology are required for their applications in a specific field. Inorganic materials are an important branch of materials, which promote development of science and technology. Alumina is an inexpensive and widely used inorganic material. It has a complex structure and many crystalline polymorphic phases such as α-Al_2_O_3_, β-Al_2_O_3_, γ-Al_2_O_3_, δ-Al_2_O_3_, θ-Al_2_O_3_, η-Al_2_O_3_, κ-Al_2_O_3_, χ-Al_2_O_3_ and ρ-Al_2_O_3_. The phase transition temperatures are different for different precursors during their calcination as shown in Fig. 1 [1].

Among the numerous crystal forms of alumina, α-Al_2_O_3_ and γ-Al_2_O_3_ are the two most common kinds. α-Al_2_O_3_ has some excellent physical and chemical properties such as good acid, alkali and heat resistances and high hardness and strength. It is widely used in different fields such as ceramics, surface protective layer materials, refractory materials, catalysts and catalyst supports and optical materials [2–5]. γ-Al_2_O_3_, which is also called activated alumina, has a large surface area, strong adsorption capacity, good catalytic activity and wear resistance. It is also widely applied in various fields such as adsorbents [6], ceramics [7], catalysts and catalyst supports [8].

The application performance of alumina depends on not only the size of ultra-fine particles but also the particle shape [9–11]. Alumina has a variety of shapes such as rod [12], fibrous structure [13], flake [14] and sphere [15]. Different shapes of alumina have different physical and chemical properties and applications. For example, the fibrous nano-alumina has a very strong anti-sintering property [16], which is often used as an additive for epoxy resin to improve its tensile strength and rigidity. The flake-like alumina is generally used as a seed crystal added to ceramics, which significantly enhances the toughness of ceramics [17].

Alumina is a common catalyst support, whose pore structure is closely related to the activity, selectivity and lifetime of the catalyst. Alumina is divided into different categories such as microporous alumina, mesoporous alumina and macroporous alumina according to its pore size. The pore size of mesoporous alumina is between 2 and 50 nm. It is a rigid porous material with a mutually interconnected or isolated network structure. It has not only the characteristics of a crystalline phase of alumina but also the characteristics of a porous material. Mesoporous alumina is widely used in the catalysis [18], adsorption [19] and other fields due to its adjustable pore structure, relatively large internal and external surface area and pore volume.

The morphology, purity, surface acidity and hydrothermal stability, the pore structure and other properties restrict the application of alumina. The research is ongoing on the pore structure, surface acidity and hydrothermal stability [20]. Morphology, as one of the important parameters of particle characterization, has a substantial effect on the properties and applications of the products. The morphology of particles is influenced and controlled by its crystallization habit during the preparation using liquid-phase method [21, 22], which is restricted by the environment and the growth conditions. This article reviews the research carried out on the preparation of alumina starting from the liquid-phase method for its synthesis including its mechanism and discusses the effect of different factors such as reactant concentration, temperature, pH, additives, system environment and calcination methods on the micromorphology of particles.

#### Liquid-Phase Method for Synthesis of Alumina

There are some common liquid-phase methods for synthesis of alumina, such as sol-gel method, hydrothermal method, template method, precipitation method, emulsion method or microemulsion method and electrolysis method. Alumina with different morphologies can be obtained by using different synthesis methods and optimizing the reaction conditions.

##### Hydrothermal Method

Hydrothermal method is an approach where the mixed solution is poured into a sealed reactor. Utilization of the relatively high temperature in the reactor and the high-pressure growth environment promotes the dissolution and recrystallization of poorly soluble or insoluble material. Hydrothermal methods include hydrothermal synthesis, hydrothermal treatment and hydrothermal reactions. During the hydrothermal process, the crystal grows to its largest possible size under the non-restricted conditions and its characteristics (various shapes, high degree of crystallinity, small size, uniform distribution, lighter particle agglomeration, etc.) form [23, 24]. The development of crystal face and the morphology of the crystal formed by hydrothermal synthesis are closely related to the hydrothermal conditions such as water temperature, pressure and the permittivity and viscosity and diffusion coefficient of the solution. The same type of crystal can be produced with different morphology under different hydrothermal conditions [25].

Li et al. [26] let ammonium aluminum sulfate, dispersant PEG2000 and urea disperse in deionized (DI) water and stirred them vigorously to form a solution. Then, the mixed solution was poured into a stainless steel pressure reactor with a teflon-lining. By changing the temperature of the water, mesoporous alumina with different morphologies was obtained. In the course of the reaction, the following reactions take place:

1 $$ \mathrm{C}\mathrm{O}{\left(\mathrm{N}{\mathrm{H}}_2\right)}_2+{\mathrm{H}}_2\mathrm{O}\to \mathrm{C}{\mathrm{O}}_2+2\mathrm{N}{\mathrm{H}}_3 $$

2 $$ \mathrm{N}{\mathrm{H}}_3+{\mathrm{H}}_2\mathrm{O}\to \mathrm{N}{\mathrm{H}}_4+\mathrm{O}{\mathrm{H}}^{-} $$

3 $$ \mathrm{A}{\mathrm{l}}^{3+}+3\mathrm{O}{\mathrm{H}}^{-}\to \mathrm{A}\mathrm{lOOH}+{\mathrm{H}}_2\mathrm{O} $$

**Fig. 2:**
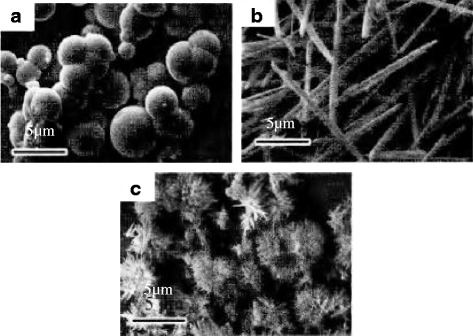
TEM image of alumina prepared under different synthetic temperatures. **a** 90 °C. **b** 120 °C. **c** 150 °C

As Fig. 2a shows, when the temperature is 90 °C, the particles obtained are different size spheres. As it is shown in Fig. 2b, at the temperature of 120 °C, the particles are superfine fiber-shaped. As Fig. 2c shows, massive fiber-shaped particles are obtained at the temperature of 150 °C. The crystal orientation is dependent on the temperature which affects the growth rate of the crystal face; consequently, the morphology can be controlled by regulating the temperature. These results indicate that the morphology of the particles substantially changes with the increasing water temperature [27].

**Fig. 3:**
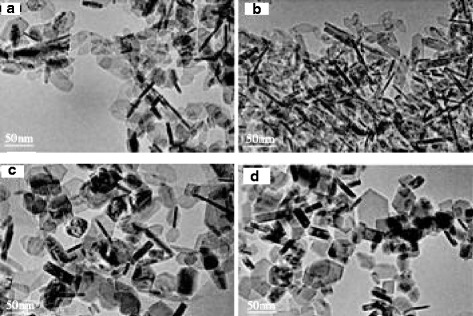
TEM image of nano-alumina with sodium nitrate concentration of **a** 0 mol, **b** 0.2 mol, **c** 0.4 mol and **d** 0.6 mol

Zhao et al. [28] prepared flat hexagonal-shaped nano-alumina by hydrothermal synthesis, using aluminum nitrate as aluminum source and sodium nitrate as additive. During the reaction, the Na^+^ of sodium nitrate was adsorbed onto the surface, which hindered the accumulation of Al^3+^ and OH^−^ ions. This affected the appearance of the particles. By changing the amount of sodium nitrate additive to control the growth of certain crystal face of alumina, hexagon-shaped alumina with different parameters was obtained. When the amount of sodium nitrate was 0.2 mol, the width of the particle was reduced and its length and thickness remained unchanged. When sodium nitrite was 0.4 and 0.6 mol, the thickness increased and the length and width remained unchanged. The hexagon-shaped particles were gradually transformed into thicker particles as the sodium nitrate concentration increased as shown in Fig. 3.

**Fig. 4:**
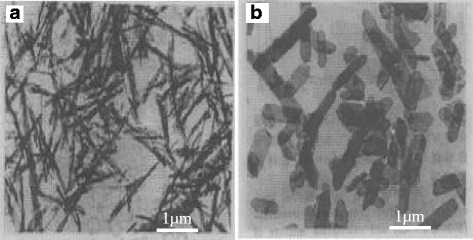
TEM image of α-Al_2_O_3_. **a** Needle-like α-Al_2_O_3_. **b** Plate-like α-Al_2_O_3_

Depending on different reaction systems, particles have accordingly different crystal habits. Pramod K. Sharma’s group and Shi’s group [29, 30] synthesized needle-like and plate-like α-Al_2_O_3_, respectively, in water and alcohol-water reaction systems by hydrothermal treatment method using Al(OH)_3_ colloid as precursor, as shown in Fig. 4.

**Fig. 5:**
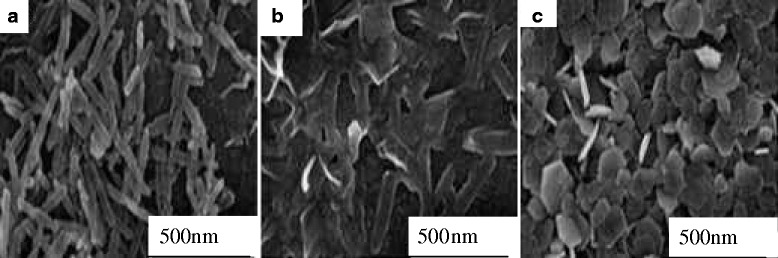
TEM image of γ-AlOOH. **a** pH = 5. **b** pH = 7. **c** pH = 9

Mikhailov et al. [31] prepared hexagonal flake-shaped γ-Al_2_O_3_ by hydrothermal method with Al_2_(SO_4_)_3_·18H_2_O and ammonia as raw materials. This study has shown that pH of solution has a significant impact on the morphology of precursor. Under acidic conditions, the H^+^ in solution will bind with the hydroxyl, which is on the surface of the γ-AlOOH layered structure, thereby destroying this structure, eventually forming a rod-like nanostructure by rolling growth mechanism [32]. On the contrary, under alkaline conditions, it retains its layered structure, forming plate-shaped nanostructure. Figure 5 shows that when pH is 5, the product is rod-like; when pH is 7, the product is transformed from rod-like to plate-shaped nanostructure; when pH is 9, the product has hexagonal shape. Boehmite converts into a γ-Al_2_O_3_ in the firing process, but its shape and size do not change [33, 34]. Calcining the plate-like precursor at 600 °C for 4 h resulted in the original hexagonal γ-Al_2_O_3_ with basically same size.

##### Sol-Gel Method

The sol-gel method refers to inorganic or organic alkoxide dispersed in solution. Using the transparent sol formed by hydrolysis and condensation of the precursor, a gel with certain structure is formed during the aging process by the aggregation between the gel particles. During the sol-gel process, the microstructure of the material is controlled and cut at the mesoscopic level by means of low-temperature chemical method, which changes the morphology and structure of the particles [35, 36].

**Fig. 6:**
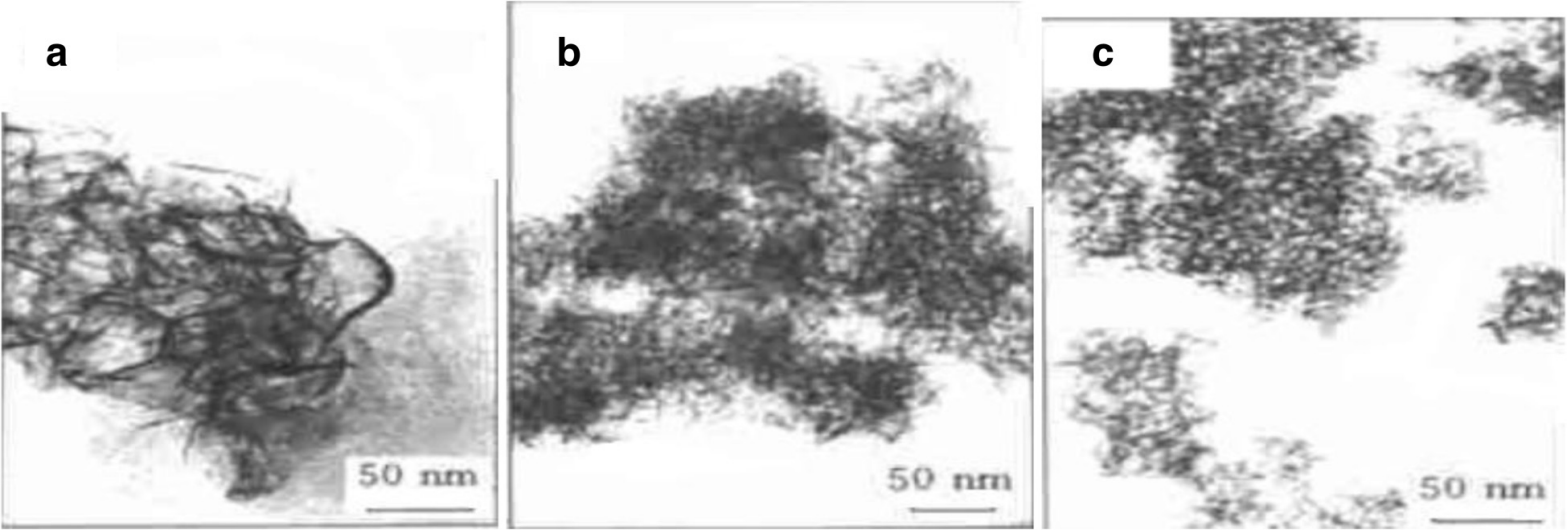
TEM image of alumina nanoparticles with different amount of AcOH. **a** No AcOH. **b** m(AcOH)/m[Al(Opri)_3_] < 0.05. **c** m(AcOH)/m[Al(Opri)_3_]≈0.1

Ning et al. [37], using AcOH as additive and adopting two-step alkoxide hydrolysis sol-gel method, synthesized spherical and fibrillar Al_2_O_3_ nano-powder in organic phase. The results showed that the amount of AcOH has a decisive effect on the morphology of the particles. As the amount of AcOH increased, the shape of the particles gradually shifted from the fibrillar to the spherical shape, as shown in Fig. 6. During the reaction, AcOH and other organic molecules containing functional groups N, O and S (ethylacetoacetate, polyamide carboxylic acid salt) as additives coordinate with inorganic ion or are adsorbed onto the surface of crystal nucleus, which changes the growth rate of crystal face. This leads to the change in the morphology of particles.

**Fig. 7:**
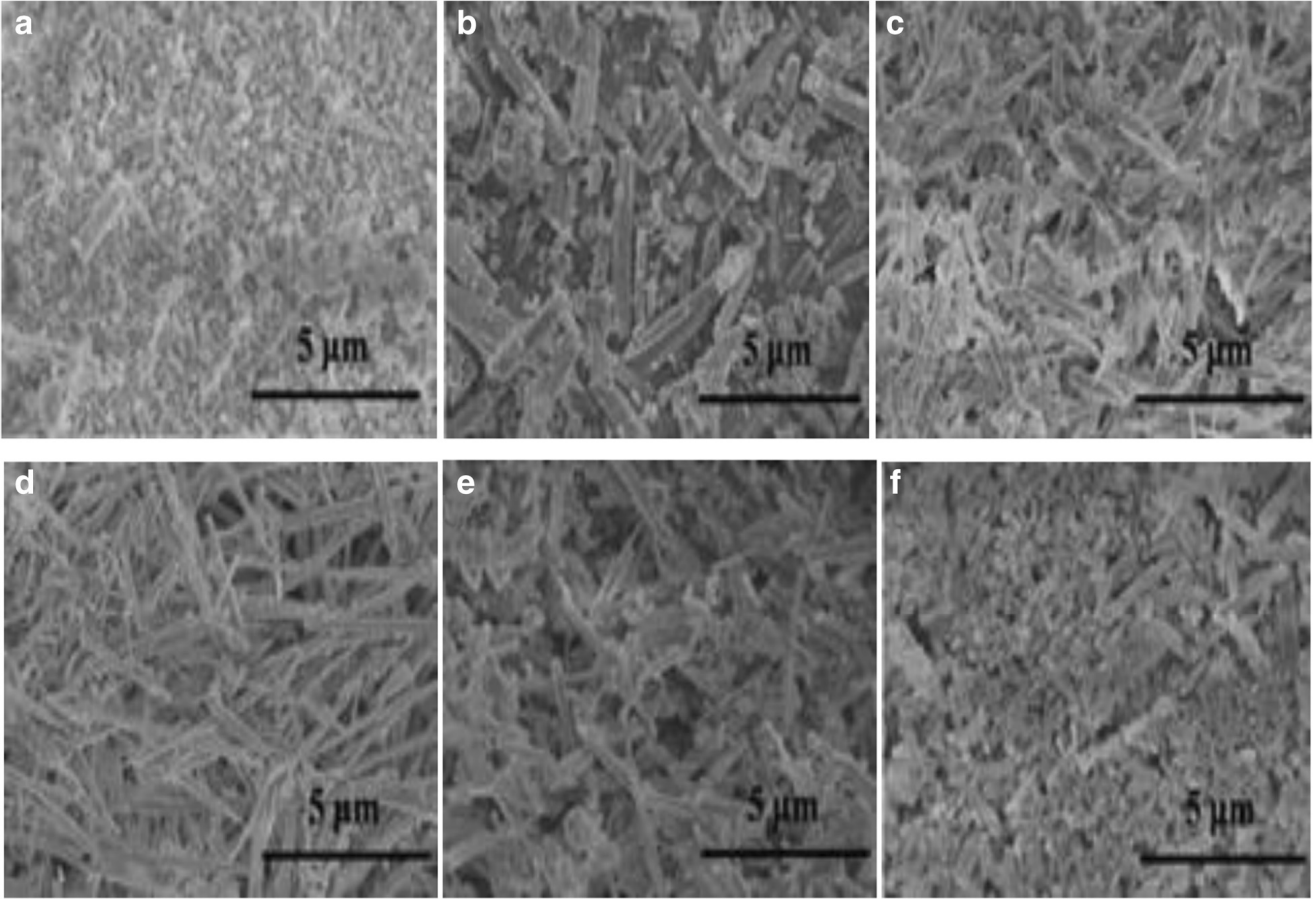
SEM image of alumina particles with different molar ratio of [Bmin]PF_6_ and aluminum isopropoxide. **a** Al_2_O_3_-0. **b** Al_2_O_3_-0.03. **c** Al_2_O_3_-0.12. **d** Al_2_O_3_-0.18. **e** Al_2_O_3_-0.24. **f** Al_2_O_3_-0.30

Masouleh’s group and Ji’ group [38, 39], using aluminum isopropoxide as aluminum source, [Bmim] PF6 as ionic liquid and adopting sol-gel method to change the molar ratio of [Bmim] PF6 and aluminum isopropoxide, successfully synthesized uniform rod-like mesoporous γ-Al_2_O_3_. This study has shown that ionic liquid plays a very important role in the morphology of products. As it is shown in Fig. 7, with the molar ratio of [Bmim]PF6, aluminum isopropoxide increases from 0 to 0.18; the morphology of the products shows a highly homogeneous rod shape. When the molar ratio of [Bmim]PF6 and aluminum isopropoxide is 0.18, the morphology of the products with rod shape has the best homogeneity. If this ratio exceeds 0.18, it is not conducive enough to form the rod shape.

##### Template Method

Template method is a cutting-edge technology developed in the 1990s. It is widely applied in recent years, and it is an effective synthetic method for controlling the structure, particle size and morphology of materials through a utilization of template. Depending on the differences in template structure, template method can be divided into two groups called hard and soft template methods.

##### Hard Template

In hard template method, precursor is uniformly dispersed in the pore of the hard template or absorbed onto its surface, thereby converting it into a complex product. Then, by choosing an appropriate method (dissolution, sintering, etching, etc.), the target product can be obtained. The special structure of hard template restricts the crystallization or polymerization of the precursor during the process of synthesis, which leads to formation of a mesoscopic phase with an opposite-phase structure of the template.

The hard template is often used as a microreactor during the synthesis. The type of hard template and the reaction conditions such as concentration of reactants, time of immersion, temperature of immersion and the temperature of heat treatment affect the structure and morphology of the product. Especially, the temperature of heat treatment has a great impact on product. The excessively high temperature causes microscopic particles to gather together which in turn affects the order of the micromorphology and its structure [40].

**Fig. 8:**
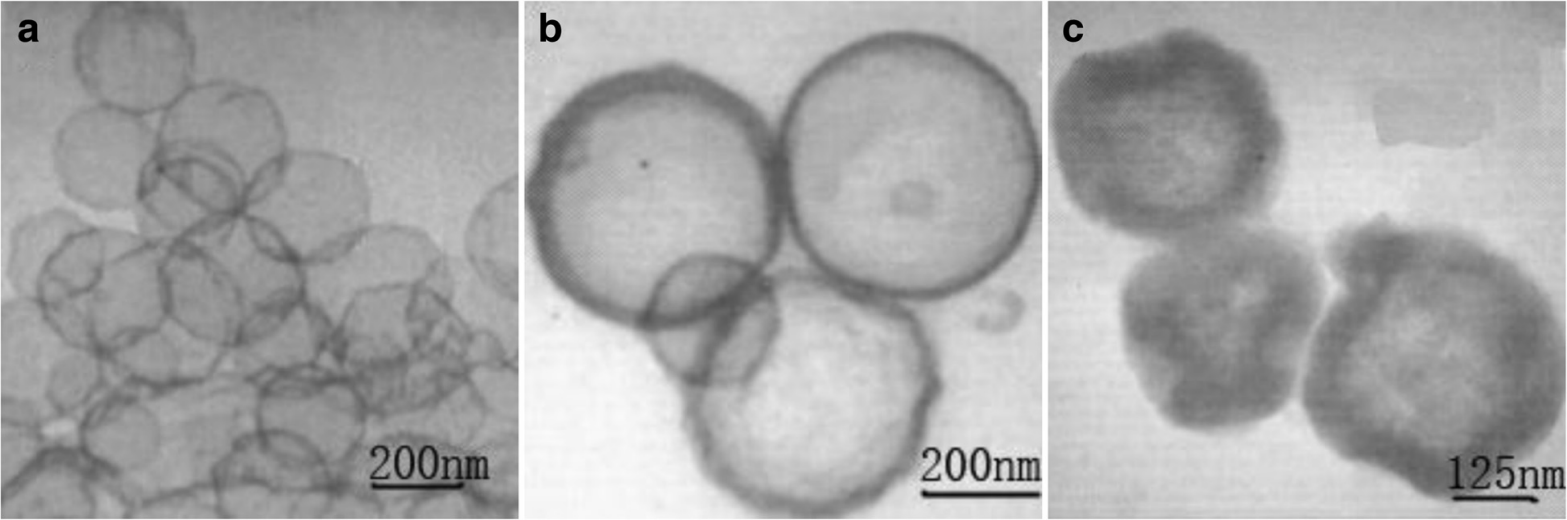
TEM image of alumina bubble with different synthetic temperatures. **a** 25 °C **b** 45 °C and **c** 55 °C

Pang et al. [41] successfully prepared alumina bubble with tunable pore size using colloidal carbon spheres as template and aluminum nitrate as the aluminum source as shown in Fig. 8a. This study has shown that the concentration of aluminum nitrate has no significant effect on the morphology and pore size of alumina. Also, the adsorption time does not affect the morphology; however, the pore diameter increases gradually with increasing time. The adsorption temperature as well has an effect on the morphology. The surface of the particles becomes smooth, and the wall thickness increases with increasing temperature as shown in Fig. 8b, c.

##### Soft Template Method

Soft template utilizes the intermolecular or intramolecular interaction forces, such as hydrogen bonds and bond and static electricity, to form aggregates with certain structural characteristics (liquid crystal, vesicles, micelle, microemulsion, self-assembled film, etc.) during the reaction. The reactants use these aggregates as template to generate a particle with certain morphology and structural features.

In the synthesis by soft template method, it is usually thought that the interaction between liquid crystalline phase and organic/inorganic interface plays a decisive role in the morphology of mesoporous materials [42, 43]. The liquid crystalline phase formed by the surfactant in solution has a rich structure such as lamellar phase, cubic phase and hexagonal phase and is easy to construct and adjust [44]. The interaction of the organic/inorganic interface is a weak hydrogen bond force in the strong acid environment while it is a strong electrostatic attraction force in the strong alkaline environment [45].

**Fig. 9:**
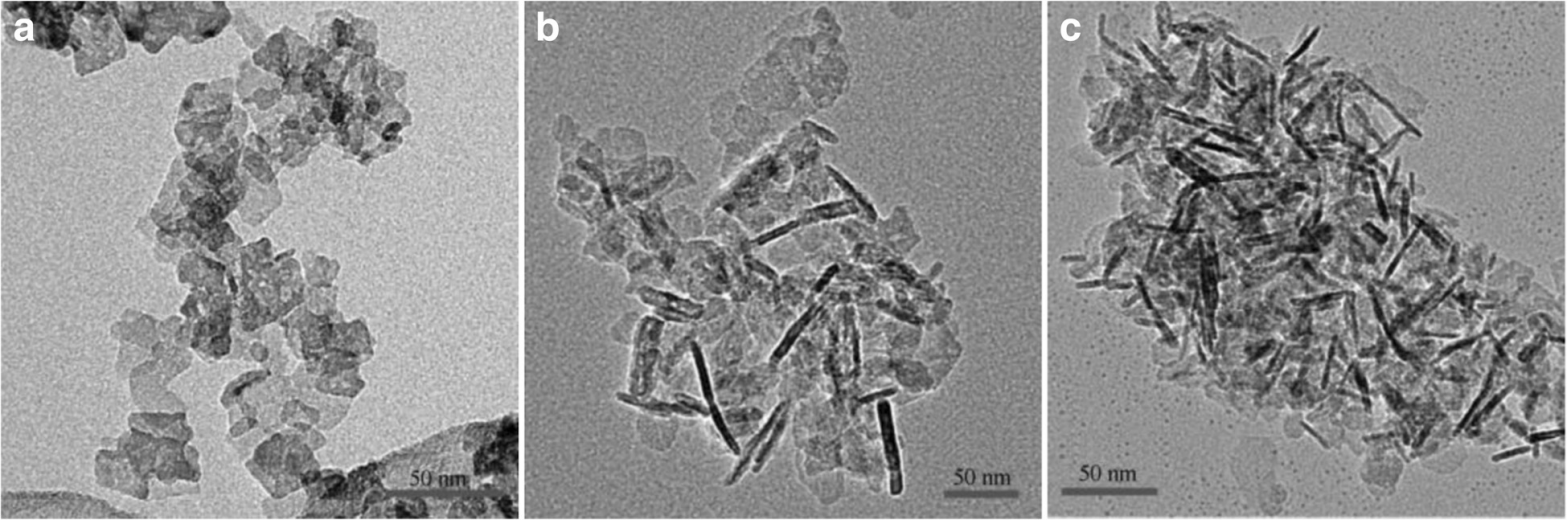
TEM image of alumina with different morphology. **a** Al_2_O_3_-∞. **b** Al_2_O_3_-60. **c** Al_2_O_3_-30

**Fig. 10:**
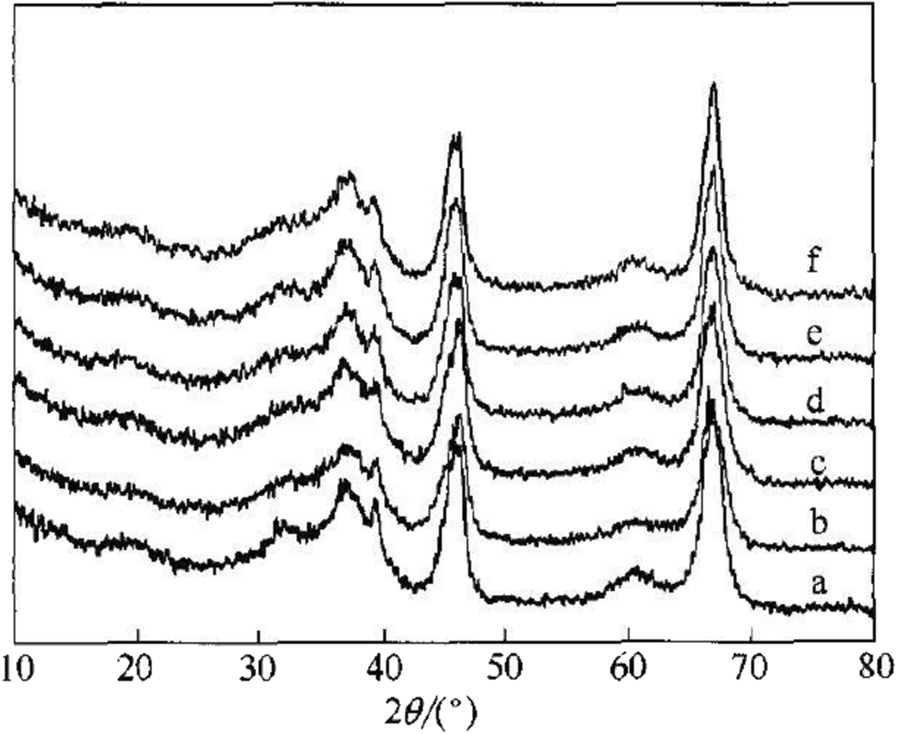
Wide-angle XRD patterns of alumina synthesized with different F127 molar ratio. *a* Al_2_O_3_-∞, *b* Al_2_O_3_-1500, *c* Al_2_O_3_-1000, *d* Al_2_O_3_-500, *e* Al_2_O_3_-60, *f* Al_2_O_3_-30

Gu et al. [46] successfully synthesized plate-like and rod-like mesoporous alumina, using F127 as soft template and aluminum isopropoxide as alumina source, and changing the mole ratio of aluminum isopropoxide and F127, as shown in Fig. 9. This study showed that the molar ratio of aluminum isopropoxide and F127 has an obvious effect on the morphology of the product. It is gradually transformed from square to plate and rod, and eventually, all become rod-like as the molar ratio increases. The result of crystalline phase analysis showed the diffraction peaks that are indexed at (311), (222), (400) and (440) associated with γ-alumina which become wider from curve a to f (Fig. 10). This XRD results suggest that the crystallite size can be smaller by increasing of F127 amount. Soft template F127 gives a good performance to weaken the crystallization process.

Groenewolt et al. [47] synthesized the ordered mesoporous γ-Al_2_O_3_ by using the soft template method. They have systematically studied the effects of various factors such as the type of aluminum source, the type of surfactant, the type of the acidity regulator and the reaction temperature on the structure and morphology of the products.

##### Precipitation Method

Precipitation method produces the target products by adding the precipitant agent to the metal solution and heat treating the precipitate. The particles with different morphology can be obtained by adjusting the reaction temperature, the concentration of the reaction, pH, etc.

**Fig. 11:**
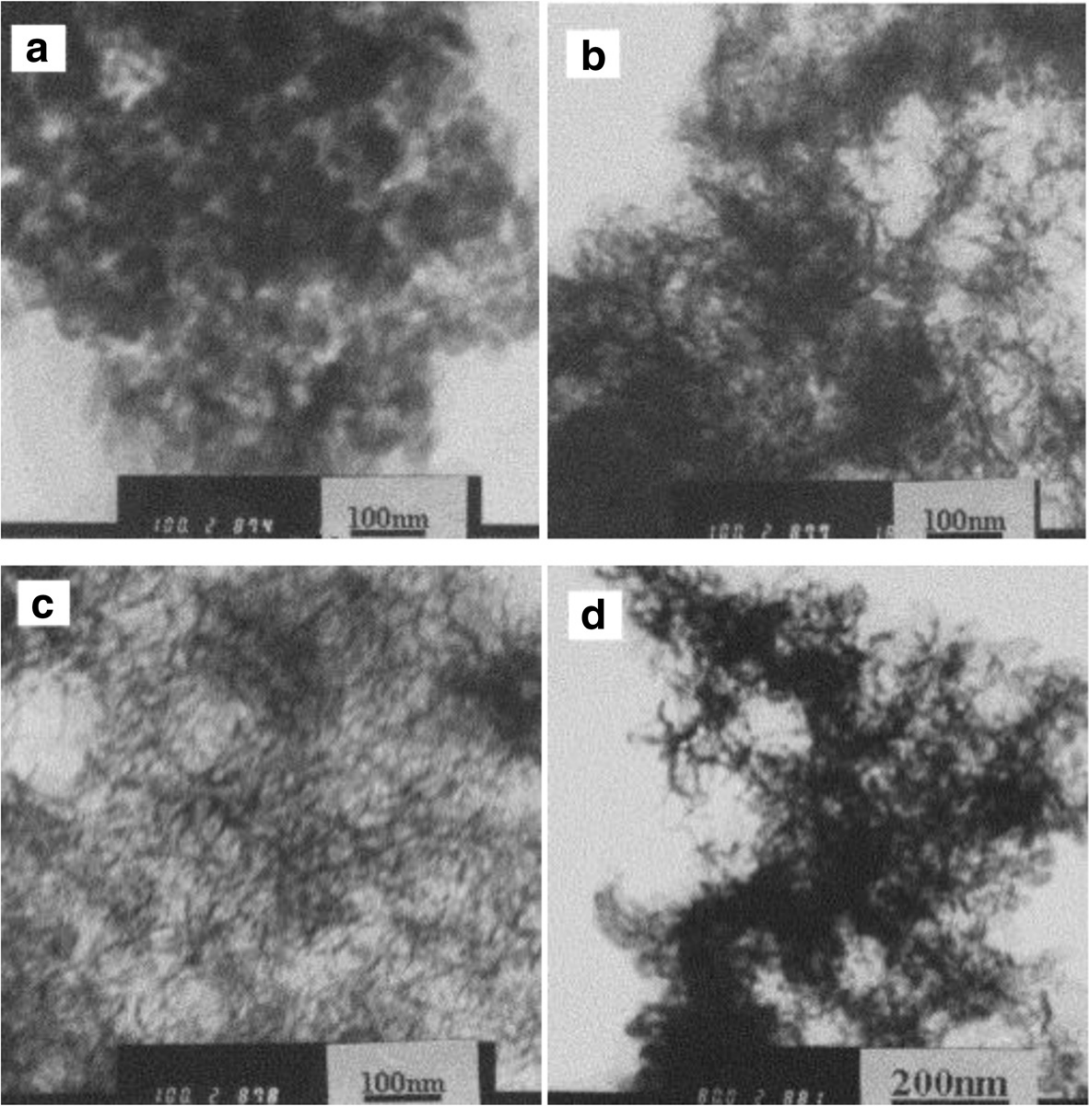
TEM image of alumina with different synthetic temperatures. **a** 40 °C. **b** 55 °C. **c** 65 °C. **d** 80 °C

Zhou et al. [48] synthesized fibrillar nano-Al_2_O_3_, using Al_2_(SO_4_)_3_·18H_2_O and NaOH as raw materials with direct precipitation method. They discussed, respectively, the effect of reaction temperature and the concentration of Al_2_(SO_4_)_3_ on the morphology (Fig. 11). The results showed that the fibrillar nano-Al_2_O_3_ with good dispersion was obtained at 65 °C.

**Fig. 12:**
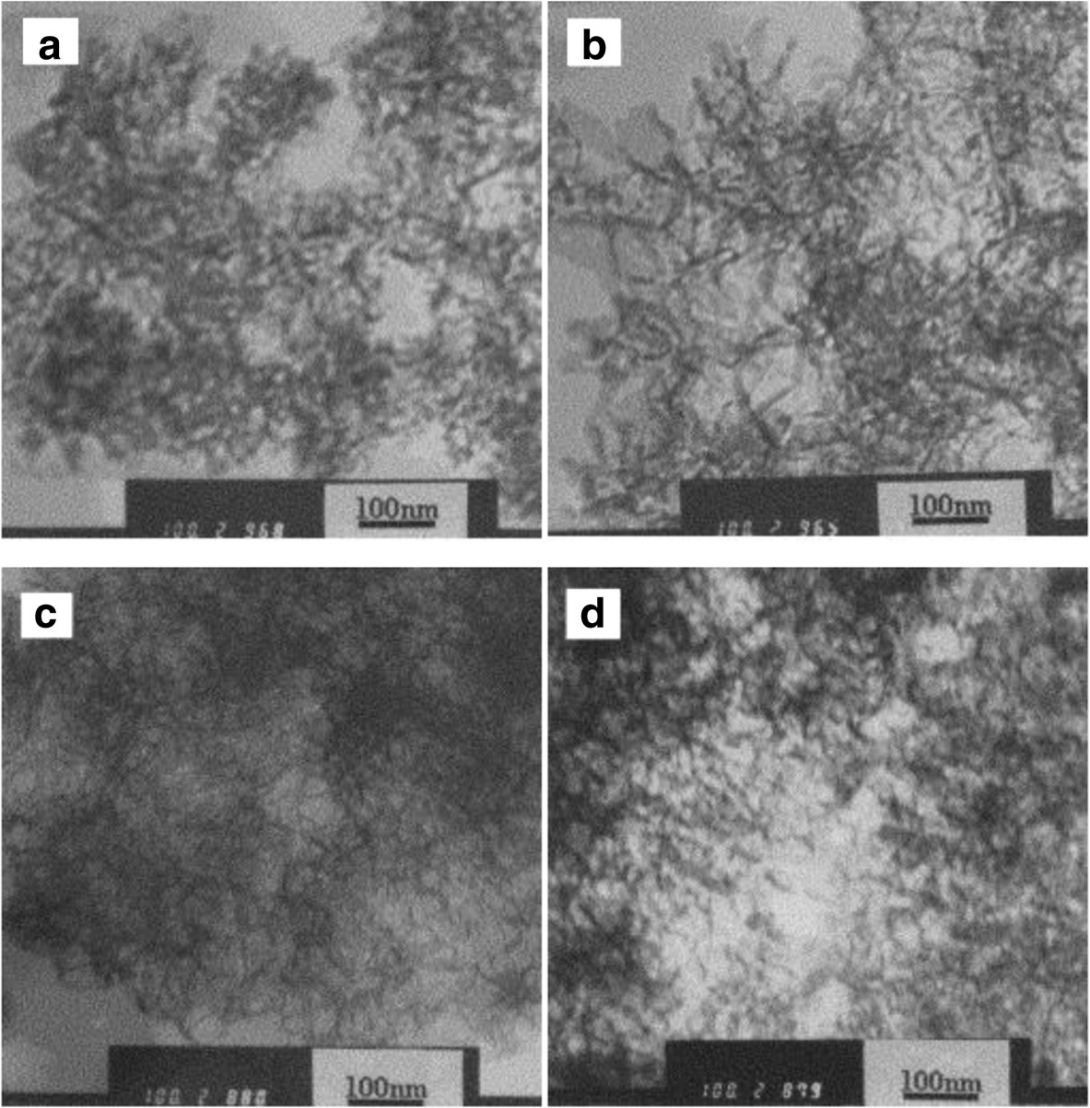
TEM image of alumina of different concentration. **a** 1.0 mol L^−1^. **b** 0.8 mol L^−1^. **c** 0.5 mol L^−1^. **d** 0.3 mol L^−1^

The concentration of reactants is also one of the important factors which control the morphology and dispersion of the product. It has an effect on the formation and the growth rates of the crystal grain, and the effect on the formation rate of the grain is greater than that on the growth rate. As shown in Fig. 12, when the concentration of Al^3+^ is 1 mol L^−1^, the granular product can be obtained. When the concentration of Al^3+^ is 0.8 mol L^−1^, the fibrillar product of poor dispersion is formed. When the concentration of Al^3+^ is 0.3 and 0.5 mol L^−1^, the reticular and fibrillar products of good dispersion are formed, respectively.

#### The Effect of Calcination System on the Morphology of Alumina

The alumina calcination system is very important for obtaining nanoparticle powder with monodispersity and uniform morphology. Nano-Al_2_O_3_ powder, which is composed of widely used α-Al_2_O_3_, γ-Al_2_O_3_ and amorphous Al_2_O_3_, is generally obtained by alumina precursor calcined at different temperatures. Therefore, the compaction among alumina particles of high activity is inevitable at high temperature, which results in severe particle agglomeration and resintering of individual particles with surrounding ones after melting with a formation of dendritic structure called “neckformation” of particle [49]. The result of the experiments showed that the calcination temperature, holding time and heating rate have a significant effect on the morphology of alumina. While the temperature is less than 800 °C, alumina particles can continue to maintain their original morphology. If the temperature becomes higher than 800 °C, the activity of alumina particles is enhanced, and agglomeration begins to occur [50]. Ceresa et al. [51] first presented the relationship between temperature and phase transformation of alumina during the calcination process.

**Fig. 13:**
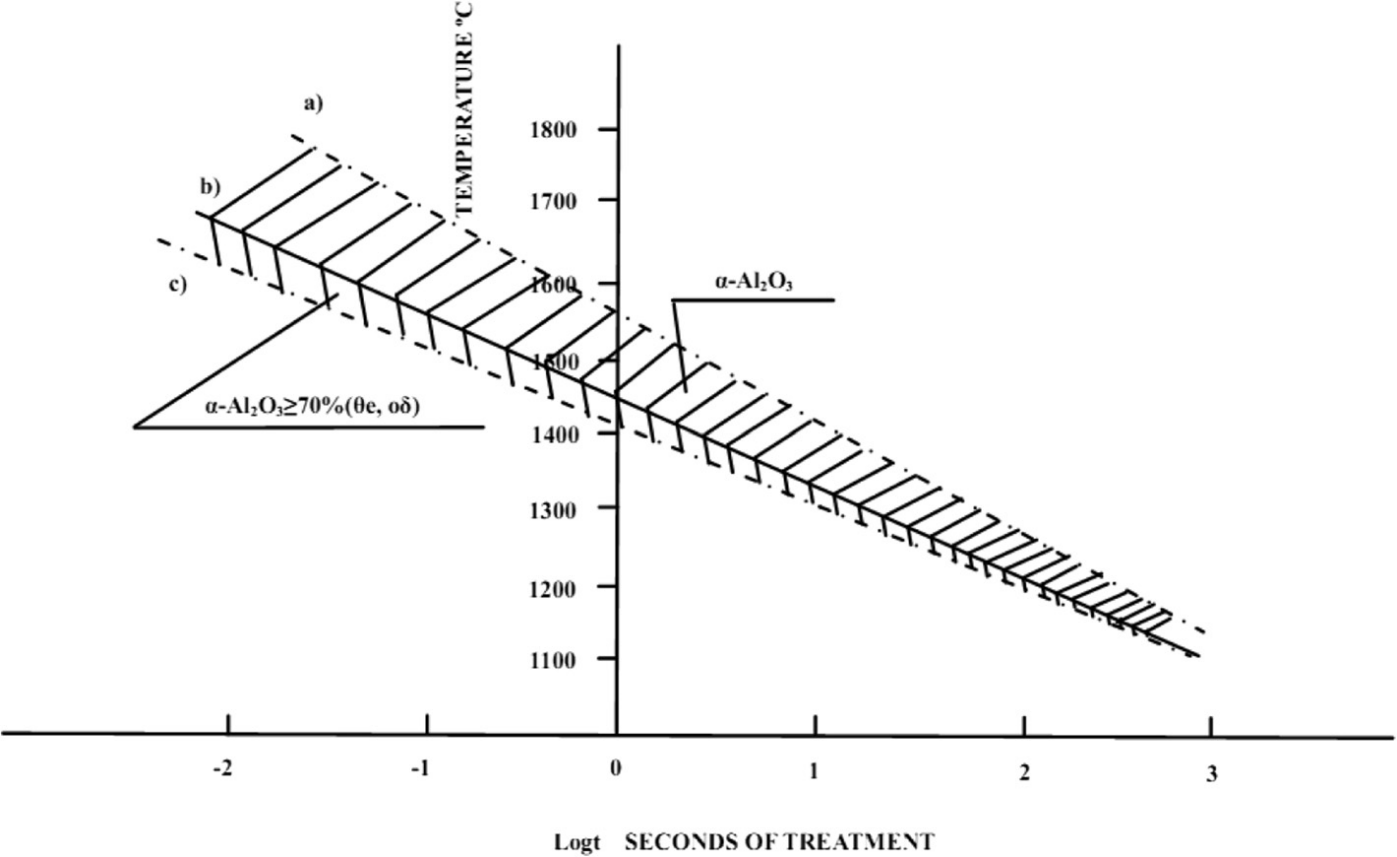
The relationship between temperature and phase transformation of alumina

It can be seen from Fig. 13 that the calcination temperature and time have a significant influence on the transformation of alumina (crystal type). When alumina particles calcined at the desired temperature in order to obtain certain crystal types, the calcination time depends on the size of the precursor. The smaller the particle size of precursor is, the shorter the time required for the calcination is, and the higher the temperature of the heat treatment is, the shorter the time required for the calcination is. The method of controlling the temperature and time during the calcination of Al_2_O_3_ is well-known. This method ensures that while the Al_2_O_3_ particles go through a complete phase change, their morphology remains unaffected and the dispersion of particles is reduced [52].

A significant amount of research is carried out in this area, and effective methods are proposed to control the morphology of alumina particles such as using DI water, alcohol and organic solvent mixtures to wash precursor before calcination in order to prevent agglomeration, enhance the dispersion, and increase the specific surface area of alumina [53]. In addition, the sintering properties of the powder can be improved with ultrasonic pretreatment, so that the neckformation created by agglomeration of the particles will not occur until 1400 °C [54]. The phase transformation temperature of γ-Al_2_O_3_ to α-Al_2_O_3_ can be decreased if sintering is carried out under the CO_2_ or ethanol atmosphere; consequently, the well-crystalline spherical α-Al_2_O_3_ is eventually obtained [55].

Dispersants and surfactants also play an effective role in dispersion of particles and control of agglomeration. For example, using poly(methacrylic acid), organic acid, glucose, sucrose, inorganic salts, trimethylsilane and other additives [56], which results in a strong electrostatic repulsion among particles, eventually change the polarity of the particle surface from hydrophilic to hydrophobic (water-repellent). Polyacrylamide, silica gel and lignin and other polymer dispersants can form a protective layer with certain strength and thickness on the particle surface and prevents the agglomeration of the particles [57]. Surfactants can form a coating layer of several nanometers on the surface of the particles, which can reduce the surface energy and effectively hinder the interactions among the particles [58].

Besides, by adding 5 wt.% α-Al_2_O_3_ seed and 44 % NH_4_NO_3_ during the calcination process, the phase transition temperature can be decreased from 1200 to 900 °C [59]. Table 1 summarizes the effects of different calcination temperatures on the grain size of Al_2_O_3_ in the presence of various additives mentioned above.

**Table 1:** Effect of calcination temperature on the size of alumina in the presence of additives

Calcination temperature (°C)	600	800	1050	1200
Al_2_O_3_ crystal type	Amorphous Al_2_O_3_	γ-Al_2_O_3_	α-Al_2_O_3_	α-Al_2_O_3_
Grain size (nm)	–	5~50	10~100	≥100
Color of Al_2_O_3_	Light yellow	White	White	White

As shown in Table 1, the amorphous Al_2_O_3_ particles obtained at 600 °C are light yellow while the additive is still present on the surface of the particles. This coating gradually disappears while 800 °C is reached. In addition, some additives can decrease the phase transition temperature of α-Al_2_O_3_ to 1000 °C. As the temperature increases, the grain size of Al_2_O_3_ will inevitably increase, meanwhile the agglomeration will start to occur. This is due to the fact that when Al_2_O_3_ completely transformed to α phase, the spatial arrangement of the O_2_ in α-Al_2_O_3_ occurs, which is the reconstruction of phase transition from face-centered cubic to hexagonal close-packed lattice [60].

## Conclusions

The morphology of Al_2_O_3_ can be influenced by various factors such as raw materials, concentrations, different synthesis methods, additives and heat treatment system. During the preparation of Al_2_O_3_, the morphology of the precursors and the protection of the particles during heat treatment play a decisive role in the final morphology of alumina. The morphology will not change during the low-temperature heat treatment. However, when high temperature is reached, the diffusion of the powder particles accelerates. Thus, the particles diffuse from the inside to the surface of the crystal lattice and spread to the surrounding resulting in the neckformation as well as the agglomeration of surrounded particles. Accordingly, the morphology of the particles changes. The use of various additives effectively reduces the calcination temperature; consequently, the problem of particle agglomeration can be solved. The utilization of template is a new research hotspot with the objective of improving the dispersibility of Al_2_O_3_ powder and controlling the shape of the sample particles.

### Prospects

In the research field of the morphology of Al_2_O_3_ and its application performance, more work is needed to obtain nano-Al_2_O_3_ powder with different shapes, single morphology and good dispersion. There is also a need to expand the application range of this type of nano-Al_2_O_3_ powder and improve its application prospect in high tech and value-added product development fields.
